# Comparing a Mobile Phone Automated System With a Paper and Email Data Collection System: Substudy Within a Randomized Controlled Trial

**DOI:** 10.2196/15284

**Published:** 2020-08-25

**Authors:** Diana M Bond, Jeremy Hammond, Antonia W Shand, Natasha Nassar

**Affiliations:** 1 Sydney School of Public Health Faculty of Medicine and Health University of Sydney Sydney Australia; 2 Strategic Ventures University of Sydney Sydney Australia; 3 Children’s Hospital at Westmead Clinical School Faculty of Medicine and Health University of Sydney Sydney Australia; 4 Department for Maternal Fetal Medicine Royal Hospital for Women Sydney Australia

**Keywords:** mobile phones, text messaging, data collection methods, clinical trial, breastfeeding, maternal health

## Abstract

**Background:**

Traditional data collection methods using paper and email are increasingly being replaced by data collection using mobile phones, although there is limited evidence evaluating the impact of mobile phone technology as part of an automated research management system on data collection and health outcomes.

**Objective:**

The aim of this study is to compare a web-based mobile phone automated system (MPAS) with a more traditional delivery and data collection system combining paper and email data collection (PEDC) in a cohort of breastfeeding women.

**Methods:**

We conducted a substudy of a randomized controlled trial in Sydney, Australia, which included women with uncomplicated term births who intended to breastfeed. Women were recruited within 72 hours of giving birth. A quasi-randomized number of women were recruited using the PEDC system, and the remainder were recruited using the MPAS. The outcomes assessed included the effectiveness of data collection, impact on study outcomes, response rate, acceptability, and cost analysis between the MPAS and PEDC methods.

**Results:**

Women were recruited between April 2015 and December 2016. The analysis included 555 women: 471 using the MPAS and 84 using the PEDC. There were no differences in clinical outcomes between the 2 groups. At the end of the 8-week treatment phase, the MPAS group showed an increased response rate compared with the PEDC group (56% vs 37%; *P*<.001), which was also seen at the 2-, 6-, and 12-month follow-ups. At the 2-month follow-up, the MPAS participants also showed an increased rate of self-reported treatment compliance (70% vs 56%; *P*<.001) and a higher recommendation rate for future use (95% vs 64%; *P*<.001) as compared with the PEDC group. The cost analysis between the 2 groups was comparable.

**Conclusions:**

MPAS is an effective and acceptable method for improving the overall management, treatment compliance, and methodological quality of clinical research to ensure the validity and reliability of findings.

## Introduction

### Background

Participant engagement and response is a vital aspect of any clinical research study. Many research studies are costly, labor intensive, and potentially compromised because of the difficulties associated with patient compliance, engagement, incomplete data collection, and inadequate follow-up [1-3]. The method and type of data collection system utilized to recruit participants and collect data throughout the study is important to ensure the quality, reliability, and validity of data collection. In addition, it must be cost-effective and acceptable to participants, funding organizations, and researchers [4-6].

Paper-based data collection in research studies is gradually being replaced or used in conjunction with electronic data collection systems [7], primarily in the form of emails containing links to web-based surveys. Comparison of these two methods has been well documented [8-11].

In recent years, mobile phone technology has been increasingly used to promote health-related behavioral change and self-management of care via the use of apps and automated SMS text messages. Studies have shown effective changes in psychological and physical symptoms [12-14] as well as specific pregnancy and breastfeeding outcomes [15,16] by sending individually tailored text messages to participants. However, a Cochrane review specifically looking at mobile phone apps as a method of data delivery for self-administered questionnaires found that none of the included trials in the review reported data accuracy or response rates [17]. Furthermore, a review of studies utilizing mobile phones for data collection showed that they were based on very small sample sizes, collected intermittent data (as opposed to daily), or had limited longitudinal data collection (maximum 9 months) [18-21]. There is also limited assessment of mobile phone technology as part of a web-based automated system, integrating randomization, SMS delivery, and electronic data collection into a streamlined data management system. Although previous studies have compared traditional paper-based data collection with data collection using mobile phones [22,23], there is limited evidence assessing the effectiveness of a combination of paper or email-based methods in comparison with mobile phones as part of an automated data collection management system. In addition, longitudinal data collection using mobile phone technology has not been assessed, particularly in maternal and infant health, despite adults of reproductive age currently being the largest users of mobile phones [24].

### Objectives

The primary aims of this study were to compare a web-based research management system utilizing mobile phone technology with a traditional delivery and data collection system using a combination of paper- and email-based methods on clinical research outcomes and to assess the acceptability and effectiveness of use, including cost analysis.

## Methods

### Design

We conducted a prespecified substudy as part of the APProve (C*A*n *P*robiotics Im*Prove* Breastfeeding Outcomes?) trial to compare a mobile phone automated system (MPAS) with a paper and email data collection (PEDC) system. APProve was a double-blind randomized controlled trial (RCT) evaluating the effectiveness of an oral probiotic versus a placebo for preventing mastitis in breastfeeding women. It was conducted between April 2015 and December 2016 in 3 maternity hospitals in Sydney, Australia. Detailed methods have been published previously [25]. Briefly, it involved the evaluation of a probiotic versus a placebo taken daily for 8 weeks for the prevention of mastitis, which was assessed using short daily and slightly longer weekly questionnaires during the first 8 weeks following birth and longer follow-up questionnaires at 2, 6, and 12 months.

The MPAS was a data delivery and collection system that combined treatment randomization, SMS delivery to participants, electronic data collection, and data management. It was developed by the study team with the aid of an eResearch (electronic research) company, which developed the system based on our prospective design specifications. The system integrated 2 established software services, SMS delivery and a web-based survey tool, which were then linked to a secure web-based data management system. The MPAS sent automated text messages to the participants’ mobile phones with links to self-administered web-based surveys. Each survey link was embedded with the participant’s unique identifier, enabling comparison across multiple surveys. A maximum of 2 automated reminders were integrated into the system if a participant did not respond after 3 days. The MPAS was pilot tested by 17 members of the research department, with feedback and suggestions integrated into the system before study commencement.

The PEDC included a combination of an 8-week calendar diary provided to participants at the time of trial entry and emailed links to weekly and follow-up surveys. The calendar diaries were identified with the participant study number at the time of treatment randomization, and the start date was manually entered. The A4-size calendar was preserved with a waterproof coating, allowing for daily entries by pen. Participants were encouraged to hang the calendar in a prominent place at home. PEDC users were supplied with a stamped, addressed envelope to post the calendar back to the trial coordinating center at the end of the treatment phase.

The study was approved by the Northern Sydney Local Health District Human Research Ethics Committee, approval number HREC/14/HAWKE/358, and registered with the Australian New Zealand Clinical Trials Registry, registration number ACTRN12615000923561. Written informed consent was obtained from all participants.

### Participants and Study Procedures

Of the 639 women randomized to the APProve trial, 539 women were allocated to the MPAS and 100 women to the PEDC. A quasi-randomization process was applied for PEDC recruitment, which was conducted on randomly preassigned days of the week and continued until 100 participants were recruited. Both groups of women were identified, approached, and consented to the study in the postnatal ward in the same way, but the treatment randomization process was slightly different.

For the women allocated to the MPAS group, a research assistant entered their details into the web-based data management system, which then automatically generated a unique participant identification number and treatment allocation. The randomization schedule was built into the system and generated using a computer random number generator with random block sizes. Randomization of participants using the PEDC was conducted using sealed, opaque envelopes, with the randomization schedule developed using a similar but separate process compared with the MPAS group.

### Data Collection

Baseline sociodemographic, clinical, and birth characteristics collected in this study are shown in Table 1. All daily, weekly, and follow-up questionnaires were identical for the 2 groups.

**Table 1:** Characteristics of participants using the mobile phone automated system compared with the paper and email data collection system.

Participant characteristics			MPAS^a^ (n=526)	PEDC^b^ (n=94)	Statistics^c^			*P* value
					*t* value (*df*)	Chi-square (*df*)		
**Maternal**								
	Maternal age (years), mean (SD)		33.4 (4.9)	33.5 (4.0)	0.06 (618)	N/A^d^	.95	
	Born in Australia, n (%)		256 (48.7)	56 (59.6)	N/A	3.8 (1)	.05	
	**Ethnicity, n (%)**				N/A	0.2 (2)	.92	
		Asian	110 (20.9)	19 (20.2)	N/A	N/A	N/A	
		White	365 (69.4)	67 (71.3)	N/A	N/A	N/A	
		Other	51 (9.7)	8 (8.5)	N/A	N/A	N/A	
	Tertiary education^e^, n (%)		440 (83.7)	77 (81.9)	N/A	0.2 (1)	.68	
	Alcohol in pregnancy, n (%)		58 (11.0)	11 (11.7)	N/A	0.0 (1)	.85	
	First baby, n (%)		312 (59.3)	44 (46.8)	N/A	5.1 (1)	.02	
	Allocated to probiotic, n (%)		265 (50.4)	46 (48.9)	N/A	0.1 (1)	.80	
**Birth, infant, and postpartum**								
	Caesarean section, n (%)		163 (31.0)	25 (26.6)	N/A	0.7 (1)	.39	
	Birthweight (grams), mean (SD)		3421 (458.1)	3456 (451.6)	0.69 (618)	N/A	.49	

^a^MPAS: mobile phone automated system.

^b^PEDC: paper and email data collection.

^c^Test statistics using Pearson chi-square test for categorical variables and 2-tailed, independent sample *t* test for continuous variables with their respective *df* are presented.

^d^N/A: not applicable.

^e^College, university, or vocational training after high school.

For the MPAS group, each study site was provided with an electronic tablet with internet connectivity to enable the research assistant to enter the participants’ details, conduct treatment randomization, and enter baseline and hospital data directly into the web-based data management system. All research assistants were trained in the use of the MPAS and given individualized password-protected access to the website, which could be accessed by phone, tablet, or computer. Only deidentified data were entered into the database and linked to an individual study number generated automatically at randomization. The only paper-based data for this cohort included a signed patient information and consent form and a trial entry form containing the participants’ contact details. Once randomized, the study number generated by the MPAS was written in the trial entry form to allow for reidentification, if required. An audit trail was integrated into the MPAS to log all SMS messages sent and surveys completed. Daily and weekly outcome data for the APProve trial for the first 8 weeks (56 days) following birth were collected via self-completed questionnaires using automated weblinks sent directly via SMS to the participant’s mobile phone. Before the follow-up questionnaires at 2, 6, and 12 months (63, 180, and 360 days), participants were sent an automated link asking for their preferred method of receiving the questionnaires, with SMS, email, or post as options. On the basis of the response, the MPAS would either send the participant an SMS link to the relevant survey or alert the trial coordinator by an automated email of the preference for an emailed or a postal questionnaire.

**Figure 1:**
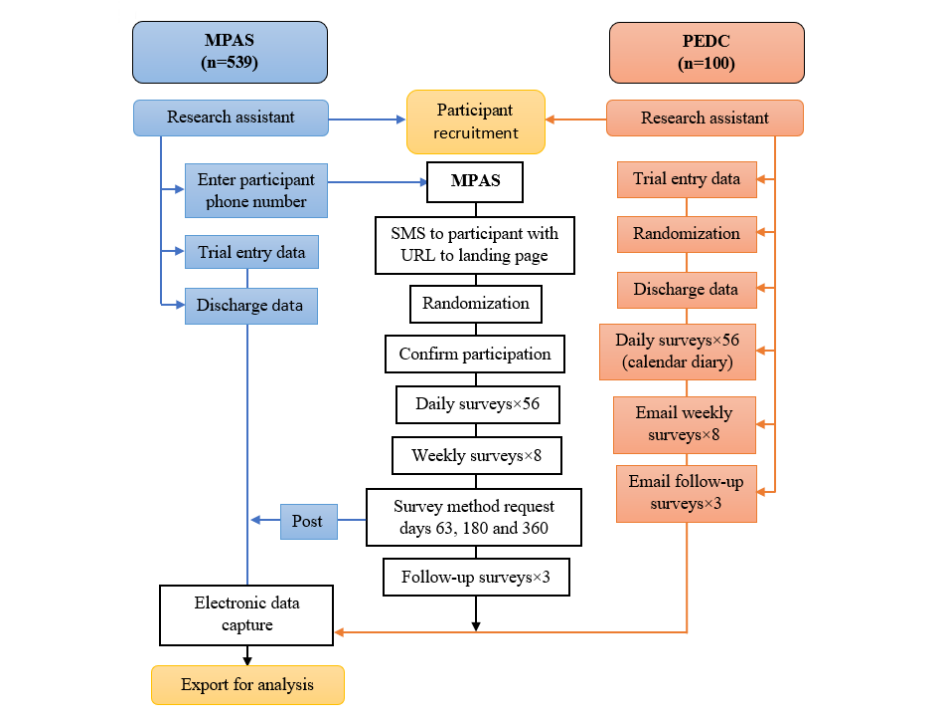
Flow diagram comparing the mobile phone automated system with paper and email data collection. MPAS: mobile phone automated system; PEDC: paper and email data collection.

For the PEDC participants, baseline and hospital data were collected on paper-based data forms and then entered into the web-based system at the trial coordinating center. Once randomized to their allocated treatment, participants were given a calendar diary by the research assistant to record daily outcomes for 8 weeks. Weekly outcome data for the first 8 weeks and follow-up questionnaires at 2, 6, and 12 months were collected by an emailed weblink to a web-based survey sent by the clinical trial coordinator (Figure 1).

### Outcomes

Outcomes evaluating participant acceptability, treatment compliance, and effectiveness of data collection comparing the MPAS with the PEDC were assessed in the 2-month follow-up questionnaire. Data were collected on the ease of participation in the trial and the ease of remembering to take the study treatment every day (both rated from 0 [very difficult] to 5 [very easy]), self-reported compliance with taking the allocated treatment (compliance was defined as having taken the product for ≥42 of 56 days, semicompliance as having taken the product for 15-41 of 56 days, and noncompliance as having taken the product for ≤14 of 56 days), whether the method of data collection was helpful in reminding the participant to take the treatment (ranked from 0 [not helpful at all] to 5 [very helpful]), recommendation of the allocated method of data collection for future studies, and the preference for how the participant wanted to receive the follow-up questionnaires (SMS, email, or post). The effectiveness of data collection was defined as the frequency of completing the questionnaires at all time points.

We also assessed whether the data collection method had any impact on the clinical trial outcomes. Clinical outcomes were collected during the daily, weekly, and 2-month surveys. They included mastitis, maternal infection, and breastfeeding status up to 2 months after birth. The mastitis outcome measure was based on self-reported symptoms related to breast infection or a clinical diagnosis of mastitis by a care provider [26].

Satisfaction with using their assigned method of data collection (MPAS or PEDC) was assessed by using open-ended free text questions to elicit written comments pertaining to what the participants liked the most and the least about their assigned method of data collection and what suggestions could be provided for future use. In addition, satisfaction with the method of data collection was elicited from the MPAS users and responses ranked from 0 (did not like at all) to 5 (really liked it). This response was subgrouped into 2 categories: *satisfied* (4-5) and *less satisfied* (0-3).

The cost analysis of utilizing the MPAS compared with the PEDC was also performed. Costs included those associated with the initial development and ongoing usage of each system and personnel time associated with trial participant survey collection and follow-up. A web-based time tracking report was generated weekly to determine the average time required for creating and sending emails and manual data entry from paper survey collection.

### Statistical Analysis

Baseline sociodemographic, clinical, and birth characteristics were compared between the 2 groups. Categorical data were summarized using percentages, and the differences in the characteristics between the 2 groups were assessed using a chi-square test. Continuous outcomes with a normal distribution were summarized using mean and SD, and the characteristics between the 2 groups were compared using *t* tests. Data with a nonnormal distribution were summarized using medians, and the groups were compared using nonparametric Wilcoxon tests. Satisfaction with the MPAS was analyzed by maternal sociodemographic characteristics and treatment compliance. Written responses were thematically assessed by 2 authors and an external researcher, who each independently coded the data, followed by group discussion. Common themes and relevant responses were identified, and frequency was quantified. Analyses were conducted using SPSS version 24 (IBM SPSS Statistics, 2016 IBM Corporation), and *P* value <.05 was used for statistical significance.

## Results

### Participant Characteristics

Of 620 women, 526 women were quasi-randomized to the MPAS group and 94 women to the PEDC group. There were no differences between the groups except that a higher percentage of women in the MPAS group gave birth to their first baby (*P*=.02; Table 1). After loss to follow-up of 10.5% (55/526) participants in the MPAS group and 11% (10/94) in the PEDC group, secondary outcomes were analyzed for 555 women. We found no difference in the trial outcomes between the 2 data collection groups (Table 2). There was also no difference in the ease of use between the MPAS and PEDC groups. However, a higher proportion of participants using the MPAS were compliant with taking the study treatment (331/471, 70.3% vs 47/84, 56%; *P*<.001), were more likely to rate their method of data collection as being a helpful reminder to record their symptoms (median 4.37 vs 2.63; *P*<.001), and were more likely to recommend their assigned method for future use (330/349, 94.6% vs 36/56, 64%; *P*<.001). There was little difference among the characteristics of the women who were lost to follow-up compared with those for whom we had follow-up data, except that at 2 months postpartum, the former were less likely to be tertiary educated (45/65, 69% vs 472/555, 85.0%; *P*=.001).

**Table 2:** Impact and acceptability of the mobile phone automated system compared with the paper and email data collection system.

Maternal outcomes		MPAS^a^ (n=471)		PEDC^b^ (n=84)		Statistics^c^				Odds ratio (95% CI)		*P* values	
						*t* value (*df*)		Chi-square (*df*)					
Mastitis, n (%)		90 (19.1)		15 (17.9)		N/A^d^		0.1(1)		1.09 (0.59 to 1.99)		.79	
Infections (other than mastitis), n (%)		77 (16.3)		20 (23.8)		N/A		2.8 (1)		0.63 (0.36 to 1.09)		.10	
Any breastfeeding at 2 months, n (%)		443^e^ (94.5)		77 (91.7)		N/A		0.1 (1)		1.55 (0.65 to 3.69)		.32	
Exclusive breastfeeding at 2 months, n (%)		385^f^ (82.3)		67 (79.8)		N/A		0.3 (1)		1.18 (0.66 to 2.11)		.58	
Ease of participation (0-5, 5=very easy), mean (SD)		3.76 (1.31)		3.57 (1.40)		−1.02 (428)		N/A		0.19 (−0.56 to 0.18)		.31	
Ease of remembering to take product (independent of method; 0-5, 5=very easy), mean (SD)		3.21 (1.43)		2.95 (1.50)		−1.3 (427)		N/A		0.21 (−0.66 to 0.14)		.21	
**Compliant with treatment, n (%)**						N/A		15.8 (2)		N/A		<.001	
	Compliant (≥42 of 56 days)		331 (70.3)		47 (56.0)		N/A		N/A		N/A		N/A
	Semicompliant (15-41 of 56 days)		87 (18.5)		14 (16.7)		N/A		N/A		N/A		N/A
	Noncompliant (≤14 of 56 days)		53 (11.3)		23 (27.4)		N/A		N/A		N/A		N/A
Helpful reminder (data collection; 0-5, 5=very helpful), mean (SD)		4.37 (1.19)		2.63 (1.85)		−9.3 (403)		N/A		0.19 (−2.11 to −1.38)		<.001	
Recommend for future, n (%)		330 (94.6)^g^		36 (64.3)^h^		N/A		50.8 (1)		0.19 (−2.11 to −1.38)		<.001	

^a^MPAS: mobile phone automated system.

^b^PEDC: paper and email data collection.

^c^Test statistics using Pearson chi-square for categorical variables and 2-tailed, independent sample *t* test for continuous variables with their respective *df* are presented.

^d^N/A: not applicable.

^e^N=469.

^f^N=468.

^g^N=349.

^h^N=56.

### Effectiveness and Satisfaction

**Figure 2:**
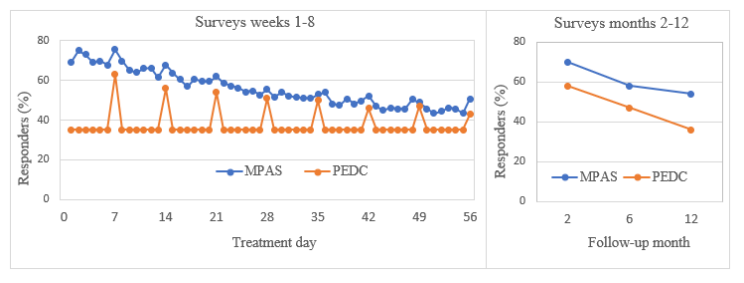
Effectiveness of data collection between the mobile phone automated system and the paper and email data collection. MPAS: mobile phone automated system; PEDC: paper and email data collection.

The frequency with which women completed the daily and weekly questionnaires was consistently higher among the MPAS users, with a 56% average response rate over the 8-week treatment period compared with 37% (*P*<.001) among the PEDC users (Figure 2). There was a gradual decrease in the MPAS daily response rate over the course of the treatment phase from 70% in the first week to less than half the women completing the questionnaires by 8 weeks. Although the daily response rate from PEDC users was lower than MPAS users, there was a notable spike in the response rate among the PEDC users on the days the weekly questionnaires were sent by email (Figure 2). Response rates for the follow-up questionnaires showed a 12% higher rate of survey completion among the MPAS users at 2 months compared with the PEDC participants, with an 18% difference at 12 months (*P*<.05; Figure 2).

**Figure 3:**
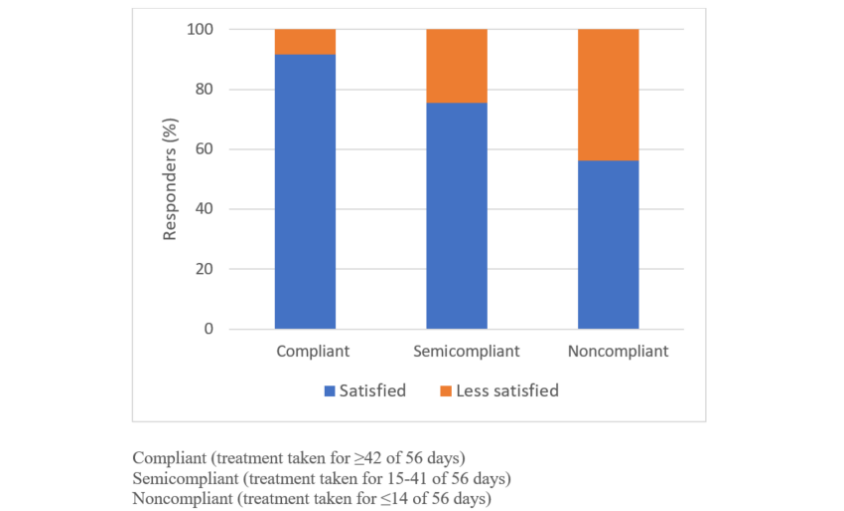
Treatment compliance and satisfaction for the mobile phone automated system (n=555).

Among the MPAS users, satisfaction was high with a mean score of 4.49 out of 5 (SD 1.0). There was no difference in satisfaction scores among maternal characteristics. There was a difference in satisfaction related to compliance, with participants most compliant with treatment being the most satisfied with the use of the MPAS (*P*<.001; Figure 3). Nearly half of the participants preferred to receive the questionnaires by either SMS (135/289, 46.7%) or email (139/289, 48.0%) at 2 months; however, the preference for SMS increased to 60% for both the 6- and 12-month questionnaires (142/241,58.9% and 135/224,60.2%, respectively). Very few women opted to receive questionnaires by post (<5%).

Responses to open-ended questions in the 2-month questionnaires were received from 74.1% (349/471) MPAS participants and 67% (56/84) PEDC participants. The themes identified were related to the factors that the participants *liked most* and *liked least* about their method of data collection as outlined in Table 3. Most of the MPAS participants stated that the MPAS was easy, convenient, quick, accessible, and efficient to use. In particular, many commented that web-based questionnaires were easy to complete while breastfeeding. Overall, less than 5% (16/349) of the MPAS participants stated that it was difficult to remember to complete the survey every day, compared with 25% (14/56) PEDC participants. Approximately, 1 in 5 participants in each group commented on the functionality of either the diary or the MPAS, such as difficulty with formatting, size restrictions, Wi-Fi accessibility, and inability to enter additional comments. Although 11 women in the MPAS group stated that they found the text messages intrusive, 3 participants stated that they liked the fact that this method was not intrusive.

**Table 3:** Qualitative analyses of the likes and dislikes of mobile phone automated system users compared with paper and email data collection system users.

Participant factors related to method of data collection		MPAS^a^ (n=349), n (%)	PEDC^b^ (n=56), n (%)
**Liked the most**			
	Ease of use	325 (93.1)	7 (12.5)
	Good reminder to take treatment	75 (21.5)	10 (17.8)
**Liked the least**			
	Nothing	168 (48.1)	10 (17.8)
	Time consuming	24 (6.9)	12 (21.4)
	Functionality issues	77 (22.1)	10 (17.8)
	Difficult to remember to complete survey	16 (4.6)	14 (25.0)

^a^MPAS: mobile phone automated system.

^b^PEDC: paper and email data collection.

Suggestions for future use by the MPAS participants included allowing users to select the time of day to receive the SMS and to opt in or out of reminder messages, limiting the number of questions on the questionnaire to minimize scrolling, diversifying the content of each SMS for improved interest, and improving the functionality to allow the questionnaires to be completed later if interrupted. Many of the PEDC participants recommended the use of SMS or a web-based app for data collection (Textbox 1).

**Participants’ comments about the mobile phone automated system compared with the paper and email data collection system.**

Mobile phone automated system

- “I found using my phone to complete the surveys great as I could do it easily when feeding my daughter.”
- “It was great—something to look forward to everyday. It was easy and also a great reminder in case I had forgotten to take my daily Approve sachets.”
- “So easy to remember and to complete the daily survey. I often completed the survey while out and about.”
- “Most people have a smartphone on hand. Much easier than using a computer or a paper record. Ease of use—always with me. Could answer questions while breastfeeding my baby.”
- “Sometimes it took a while to upload the questions”
- “Reminders were great but sometimes daily were a bit annoying”
- “Weekly questionnaires bit lengthy”
- “It would often change my response (touch feature too sensitive)”
- “Hard to see if the survey was completed if forgotten to complete the previous ones”

Paper and email data collection system

- “I liked to be a part of this study but it was not that easy to remember it to take every day...I missed sometimes.”
- “Helped to keep on track. Encouraged me to have a morning routine that incorporated having breakfast at the similar time each morning.”
- “The calendar was quick and easy. Can’t imagine also having to write in a diary on a daily basis.”
- “Now that everyone is on the phone maybe there could be a daily reminder on the participants’ phone, creating an app or site so the data goes straight to the research office daily or weekly.”
- “Filling out the manual form is troublesome.”
- “Forgetting to fill in the daily diary even though it was clearly explained to me before I agreed to do the trial. I’m so sorry. I only found it the other day in a pile of paperwork. I do everything electronically.”
- “Keeping track and filling as was not doing it every day so it was hard to remember after 15-20 days for that period, sorry.”
- “The progress chart would be easier if online or an app so it could be filled in on a smartphone during feeds.”
- “Probably use a different stock as it could be hard to write on.”

### Cost Analysis

Cost analysis between the 2 groups showed a comparable per-person cost, with the MPAS costing on average Aus $10 (US $7.21) more (Tables 4 and 5).

**Table 4:** Cost analysis for paper and email data collection

Paper and email data collection		Cost, Aus $ (US $)^a^
**Diaries**		
	Printing	854.05 (615.65)
	Labels for diaries	58.85 (42.42)
	Stamps	150 (108.13)
	Envelopes	40 (28.83)
Paper and printing (case report forms)		10 (7.21)
Emails^b,c^		5000 (3604.29)
Data collection forms		2500 (1802.14)
Reminder emails: 35% (33/94) return rate		500 (360.43)
Total×100 participants		9112.90 (6569.11)
Total cost per person		91.13 (65.69)

^a^All costs are calculated in Australian dollars (Aus $1=US $0.72).

^b^Labor is calculated at Aus $50 (US $36.04) per hour.

^c^Emails are calculated at 5 min per email.

**Table 5:** Cost analysis for mobile phone automated system.

Mobile phone automated system	Cost, Aus $ (US $)^a^
Tablets×3	1060 (764.11)
Intersect: data hosting	3300 (2378.83)
Intersect: app development	29,080 (20,962.50)
Intersect: trial Infrastructure	14,500 (10,452,40)
Web survey tool (Aus $780 per year×2)	1560 (1124.54)
SMS service (45000@Aus $0.069 per sms)	3105 (2238.26)
Mobile service number (Aus $25 per month×24)	600 (432.52)
Website hosting (Aus $25 per year×2)	50 (36.04)
Broadband (Aus $30 per month×24)	720 (519.02)
Reminder emails: 46.7% (246/526) return rate)^b,c^	450 (324.39)
Total×529 participants	54,425 (39,232.70)
Total cost per person	102.88 (74.16)

^a^All costs are calculated in Australian dollars (Aus $1=US $0.72).

^b^Labor is calculated at Aus $50 (US $36.04) per hour.

^c^Emails are calculated at 5 min per email.

## Discussion

### Principal Findings

This study demonstrates that an MPAS is an effective and acceptable tool for improving study delivery and data collection within a randomized trial as compared with a more traditional system. We have shown that the mobile phone system improved treatment compliance and response rates, demonstrated greater user satisfaction, is comparable in cost to PEDC, and does not impact study outcomes.

### Comparison With Prior Work

Our study supports previous studies which showed that SMS messaging could improve treatment adherence and was acceptable to participants [16,19,27]. Despite concerns about long-term attrition in previous studies [28], the MPAS results showed that even with a decrease in response rates over time, the response rates were consistently higher than the PEDC rates over the same period, possibly because of better engagement among the users. Although the response rate of the PEDC participants showed that 37% (35/94) of the participants returned a completed questionnaire, it is likely that some of the days may have been retrospectively completed, compromising the accuracy of the data. The peak completion rate of the PEDC questionnaires was on the day the weekly questionnaires were emailed to the participants, suggesting that emailed links are a more effective method of data collection compared with paper-based data collection, although they are more time consuming for the trial coordinator compared with automated SMS links. Despite no difference in clinical outcome measures between the 2 groups, the increased response rates to the daily surveys provided rich data regarding breastfeeding habits, confirming the feasibility of using an MPAS as a means of improving the reliability of outcome data in breastfeeding research [23].

The daily questionnaires of the MPAS appeared to have a secondary effect of improving treatment compliance by serving as a daily reminder, which in turn increased engagement with the system, resulting in a higher rate of satisfaction. Anecdotally, satisfaction among the research assistants was also high, with the majority saying that the MPAS was easy to use and less time-consuming for randomization and data entry as compared with paper forms. Moreover, the MPAS minimized the use of paper.

Despite previous research showing a 55% reduction in cost upon using electronic data collection compared with paper data collection [10], our study indicates that the cost per person is comparable between PEDC and MPAS. This is largely because of the differences in electronic data capture between the 2 studies, with the earlier study collecting, monitoring, and entering data directly into a web-based database, whereas the major expenditure to our study was the development of a research management system that integrated randomization, automated SMS, and data collection. It is important to note that once the trial infrastructure and data hosting was installed and initiated, there was potential to significantly scale up the number of participants and the duration of the study without an incremental increase in cost, whereas an increase in PEDC participants would constitute a supplemental increase in labor costs. An additional 18 PEDC participants in our study would have balanced the costs between the 2 groups. Furthermore, the scope for contact and engagement with participants with the MPAS is greater compared with paper and email methods of data collection. For example, the PEDC participants each received a minimum of 11 emails. Conversely, the MPAS participants received an average of 61 automated text messages, including welcome texts, daily SMS, and reminder messages. If the same number of texts were sent by email by a clinical trial coordinator, the cost would have increased to an additional Aus $200 (US $144.17) per participant (Aus $292 [US $210.49] PEDC vs Aus $102 [US $73.53] MPAS).

There is very little data to evaluate the use of SMS as a consolidated research management tool. We found many benefits of using MPAS in the multicenter APProve trial, including a centralized system to manage randomization, data collection across all stages of the trial, automated reminders and alerts, reduced paper transfer of sensitive patient information between sites, reduced potential for transcription error [11,29,30], and improved reliability of daily data collection associated with reduced risk of recall bias [23]. Reducing the burden and time of data collection on the research assistant was significant, along with issues associated with patient confidentiality and storage of physical case report forms [23,29]. The advantage of integrating the MPAS via a web-based platform ensured access across mobile phone platforms and enabled accessibility to a large and diverse population, especially for those living in rural, remote, or disadvantaged areas or where mobility is restricted [31,32]. In addition, staff sick leave and absences were less of an issue because of the automated nature of the system, leading to increased flexibility of the research team, which is important when managing research studies on small budgets in small teams.

### Strengths and Limitations

The main strength of our study was embedding the assessment of the MPAS versus PEDC as a substudy in an RCT with quasi-randomization to treatment group showing little difference between study groups. Most studies comparing paper-based data collection and electronic data collection had very small sample sizes, 20 to 116 participants [20,33], whereas we were able to show an effective difference with a statistically robust sample size. Furthermore, daily data collection for 8 weeks and comparison of responses at 3 strategic time points over the course of 1 year was instrumental in the accurate assessment of outcomes and minimizing errors in recall bias [34]. The inclusion of data accuracy and response rates fills a gap in the literature as addressed by a relevant Cochrane review [17]. Furthermore, the method of data collection for both groups allowed for objectivity of responses without *gratitude bias*, as is often seen in questionnaires of a face-to-face nature [35,36].

One of the limitations of the study was the difference in sample size between the 2 groups. As this was a substudy of an RCT, it was not powered for this secondary outcome. Random sampling was performed to ensure that the MPAS did not adversely affect the primary outcome. Although baseline maternal characteristics show that more women in the MPAS group gave birth to their first baby, possibly because the paper diary appeared more overwhelming for first-time mothers, there were no differences between maternal health and breastfeeding outcomes. In addition, self-reported compliance can be perceived as subjective and prone to bias, but as compliance was measured by the same method in both groups, the bias would be nondifferential. There were also issues with the interface and usability for completing the questionnaires via the web for both MPAS and PEDC participants. However, we were able to resolve many of the issues and make slight modifications to the software over time. This did not negatively impact the response rates. A final limitation was that no assessment of participant time was included in the cost analysis. This was not included as it was not anticipated that there would have been a discernible difference in time cost between the 2 groups. Posting the diaries and logging on to the computer for the weekly questionnaires may have elicited more time from the PEDC participants, but this would have been negligible.

### Conclusions

Despite the increasing growth of web-based clinical trial management systems, there has been little or no evaluation of these systems against traditional methods of trial management systems. Since the commencement of our trial, there have been improvements in the quality and availability of electronic data collection systems. For example, REDCap (Research Electronic Data Capture) is a secure web application for building and managing web-based surveys and databases, specifically for research studies and operations [37]. The system offers an easy-to-use and secure method of flexible yet robust data collection, which is free to researchers affiliated with universities. Using such a system would have decreased the costs associated with the development of the web-based survey tool we utilized as well as eliminated many of the functionality issues we experienced to reduce future research costs.

Future research should focus on how to maximize the effect of mobile phone technology, such as implementing strategies to improve long-term engagement with participants by simplifying questionnaires, optimizing the number of text messages, and personalizing the content and timing of messages.

Although we evaluated MPAS in a perinatal population, the use of mobile phone technology provides the opportunity to facilitate and improve the quality and effectiveness of clinical research studies; enhance patient interaction; and improve clinical research across a wide range of methodologies, disciplines, and health care settings. Integration and evaluation of mobile phone research management systems that are cost-effective, efficient, and acceptable to both researchers and patients is essential, given the increasing use of mobile phone technology [24] and high costs of undertaking research. We have shown that the use of an integrated MPAS is an effective and acceptable method for improving the overall management, treatment compliance, and methodological quality of a randomized clinical trial to ensure validity and reliability of findings, in addition to being cost-effective.

## Abbreviations

APProve: CAn Probiotics ImProve Breastfeeding Outcomes?
MPAS: mobile phone automated system
PEDC: paper and email data collection
RCT: randomized controlled trial
